# The association of anxiety, depression, sleep quality and intradialytic hypotension in hemodialysis patients: a cross-sectional study

**DOI:** 10.3389/fpsyt.2026.1776422

**Published:** 2026-06-05

**Authors:** Xiaomei Jiang, Panpan Chen, Siyan Deng, Yujuan Yang, Yixiu Liu, Shuixiang Wu, Qinjuan Xu, Huagang Hu, Xiaowen Zhu

**Affiliations:** 1 Blood Purification Center, The Second Affiliated Hospital of Soochow University, Suzhou, China; 2 School of nursing, Jinan University, Guangzhou, China; 3 Jiangxi Cancer Hospital, Nanchang, China; 4 School of nursing, Suzhou Medical College of Soochow University, Suzhou, China; 5 Hemodialysis Center, The First Affiliated Hospital of Soochow University, Suzhou, China

**Keywords:** maintenance hemodialysis, intradialytic hypotension, anxiety, depression, sleep quality

## Abstract

**Background:**

Patients on maintenance hemodialysis (MHD) experience elevated rates of anxiety, depression, and poor sleep. Intradialytic hypotension (IDH) is a serious complication linked to increased mortality and hospitalization, yet its association with psychological and sleep disturbances remains unclear. This study aimed to examine the prevalence of IDH, anxiety, depression, and poor sleep in MHD patients, and to explore whether IDH is associated with these symptoms.

**Methods:**

In this cross-sectional study, 467 patients from four dialysis centers in Suzhou, China, were enrolled. IDH was assessed over 36 sessions using medical records. Anxiety (GAD-7), depression (PHQ-9), and sleep quality (PSQI) were measured.

**Results:**

Among 16,812 dialysis sessions, IDH incidence was 11.7%. A total of 123 patients (26.3%) experienced IDH frequently (≥4 episodes). Anxiety was present in 30.4% of the total sample (142/467), depression in 44.3% (207/467), and poor sleep in 39.9% (182/456). Multivariable analysis indicated that frequent IDH was significantly associated with higher anxiety, depression, and sleep quality scores (P< 0.05).

**Conclusion:**

Frequent IDH is correlated with greater symptom burden of anxiety, depression, and poor sleep quality in MHD patients. These findings highlight the need to integrate hemodynamic monitoring with psychological and sleep assessment in MHD care. Further longitudinal studies are needed to clarify causal mechanisms and evaluate interventions.

## 1 Introduction

Chronic kidney disease affects approximately 9.1% of the global population, corresponding to nearly 697.5 million individuals. Patients with end-stage renal disease (ESRD) require renal replacement therapy to sustain life, and maintenance hemodialysis (MHD) remains the predominant dialysis modality (1–5). Although MHD significantly prolong patient survival, the management of its complications remains a major clinical challenge.

Intradialytic hypotension (IDH) is a common complication during hemodialysis treatment (6). Owing to the lack of a universally accepted definition, its reported incidence varies widely across studies, ranging from 7.5% to 69% (7). According to the Kidney Disease Outcomes Quality Initiative guidelines developed by the National Kidney Foundation of the United States, IDH is defined as a decrease in systolic blood pressure ≥ 20 mmHg or a decrease in mean arterial pressure ≥ 10 mmHg, accompanied by hypotensive symptoms, and requiring clinical intervention to improve (8). Similarly, the European Best Practice Guidelines (EBPG) define IDH as a decrease in systolic blood pressure ≥ 20 mmHg accompanied by clinical events and interventions (9). However, symptoms and nursing interventions are not consistently or objectively documented in many clinical databases. Therefore, previous studies tend to use reduction in systolic blood pressure (SBP) (20, 30, and 40 mmHg) or nadir SBP below the threshold (90, 95, and 100 mmHg) to define IDH (10).

Relevant studies have confirmed that IDH can lead to physiological discomfort symptoms (e.g., dizziness, palpitations, chest tightness, etc.), and that urgent treatment or even cessation of ultrafiltration may be required to alleviate the symptoms and avoid the risk of shock (11, 12). However, there are limited evidence to verify whether these conditions influence the psychological burden of patients and are associated with the development of psychological symptoms such as anxiety and depression. In addition, poor sleep quality is a common problem in MHD patients with an incidence of 70% ~ 80% (13, 14). Previous studies have suggested that dialysis-related complications, including IDH (15), may be associated with sleep disturbances. One possible explanation is that IDH may interrupt the dialysis process, require adjustment or cessation of ultrafiltration, and potentially compromise dialysis adequacy, which has been linked to sleep quality (16–18). However, the relationship between IDH frequency and sleep quality has not been well characterized. It is hypothesized that IDH may indirectly affect the quality of sleep in MHD patients, but no studies have been conducted to clarify its intrinsic relevance.

To date, limited evidence has quantitatively examined the relationship between IDH occurrence and psychological and sleep indicators. Therefore, this study aimed to compare the differences in anxiety, depression, and sleep quality between the occasional IDH group (IDH occurrence 0 to 3 times in 36 dialysis sessions) and the frequent IDH group (IDH occurrence ≥ 4 times in 36 dialysis cases), and to explore the relationship between IDH grouping with these psychological and sleep-related outcomes.

## 2 Methods

### 2.1 Study design and participants

This cross-sectional study was conducted from April 2021 to June 2023. Data were collected from MHD patients in the outpatient hemodialysis centers of four tertiary hospitals in Suzhou City, China. A cluster sampling approach was used, with each outpatient hemodialysis center considered as a cluster. All eligible patients in the selected centers were invited to participate. Eligible MHD patients who provided written informed consent prior to study were included. Inclusion criteria (1) ESRD due to various reasons (19) and undergoing MHD; (2) age ≥ 18 years; (3) hemodialysis vintage ≥ 3 months; and (4) hemodialysis frequency 3 times a week. Exclusion criteria: (1) patients with severe infection or other systemic diseases, such as malignant tumors, autoimmune diseases, etc.; (2) pregnant or lactating patients; (3) patients with severe hearing, speech disorders, and cognitive impairment; (4) patients with fewer than 36 dialysis sessions with an available intradialytic blood pressure record within the 6 months before enrollment. This study received ethical approval from the Ethics Committee of Soochow University (Reference: SUDA20230115H02). All participants provided written informed consent, and all procedures adhered to the relevant guidelines and regulations.

### 2.2 Sample size calculation

As the prevalence of IDH among MHD patients was one of the primary outcome, the sample size was calculated using the standard formula for prevalence studies: N=[Z(1−α/2)/d] ×2p×(1−p) (20)The parameters were set as follows: α=0.05, yielding Z_(1−α/2)_=1.96, allowable margin of error(d) was set at 0.05 to ensure adequate precision. And p was based on the previously reported IDH prevalence of 29.4% (10).These parameters yielded a minimum sample size of 319 participants. To account for an anticipated 20% rate of invalid or incomplete questionnaires, the target sample size was adjusted to 399. Ultimately, 467 patients were enrolled.

### 2.3 Data collection and definition

Blood pressure and questionnaire were collected by trained investigators. Blood pressure readings were retrieved from the electronic medical record system of the blood purification center before the patient filled in the questionnaire. Starting from the previous intradialytic blood pressure on the day of filling in the questionnaire, the data were counted from recent to distant times until 36 hemodialysis sessions’ blood pressure were collected. If missing data were encountered during the process, the data were traced back up to 6 months before the questionnaire was filled out. That is, patients with less than 36 blood pressure data within 6 months before the questionnaire was filled out were not included. Considering the practical feasibility and data consistency of this study and referred to the published study (21) showing that definitions using minimum blood pressure thresholds are more strongly associated with adverse clinical outcomes, we defined IDH as the occurrence of a lowest recorded systolic blood pressure (SBP)< 100 mmHg during dialysis. The IDH grouping method was based on the study of Zhi et al. (22) and was divided into groups according to the number of IDH occurrences during dialysis: patients with 0 to 3 IDH occurrences in 36 dialysis sessions were classified as the occasional IDH group; patients with ≥ 4 IDH occurrences were classified as the frequent IDH group.

### 2.4 Variables and measures

In this study, blood pressure was measured in dialysis patients by means of a sphygmomanometer, while four instruments were used to examine the general information of the participants and to assess three dependent variables (depression, anxiety, and poor sleep quality).

#### 2.4.1 General characteristics

The general information questionnaire consists of three parts. The basic characteristics includes gender, age, height, marital status, education level, work status, average monthly income and smoking. Disease-related characteristics includes etiology of kidney disease, the number of complications other than ESRD (such as myocardial infarction, ischemic cardiomyopathy, heart failure, arrhythmia, diabetes, cerebrovascular accident, peptic ulcer, chronic obstructive pulmonary disease, mild to severe liver disease, secondary hyperparathyroidism, mineral metabolism disorder, peripheral vascular disease, hypertension, renal anemia and others), and laboratory test data (hemoglobin). Treatment-related information includes dry weight, dialysis age, dialysis method, and number of medications.

#### 2.4.2 7-items generalized anxiety disorder scale-7

The GAD-7 (23) has good reliability and validity for assessment of anxiety. The scale consists of seven items, each with the following scores: 0 = not at all, 1 = for a few days, 2 = for more than a week, 3 = almost every day, and a total score range of 0-21. A GAD-7 score of 0–4 is considered as no anxiety, 5–9 is considered mild anxiety, 10–14 is considered moderate anxiety, and 15–21 is considered severe anxiety, with higher scores indicating a more severe level of anxiety. The scale has good reliability and validity, with a sensitivity of 86.8% and a specificity of 93.4% (24).

#### 2.4.3 9-items patient health questionnaire-9

The PHQ-9 (25) has been validated as a reliable and valid screening tool to assess the frequency of depressive symptoms over the past two weeks. The scale consists of nine items on a four-point scale (0 = not at all, 1 = for a few days, 2 = for more than a week, 3 = almost every day), with a total score ranging from 0-27. A PHQ-9 score of 0–4 is considered to be no depression, a score of 5–9 is considered to be mild depression, 10–14 is considered to be moderately depressed, 15–19 is considered to be moderately severe, and 20–27 is considered to be severely depressed. The higher the score, the more severe the depressive symptoms. The scale has good reliability and validity, with a sensitivity of 88% for scores > 10, a specificity of 88%, and a positive detection rate of 31% to 51% (26).

#### 2.4.4 Pittsburgh sleep quality index

The PSQI can be used to assess the subject’s sleep quality in the last month and consists of 19 self-rated and 5 other-rated items. The 18 entries involved in the scoring of this scale make up 7 dimensions, and each dimension is scored on a 4-point scale. Very good is scored as 0, good as 1, poor as 2, and very poor as 3. The cumulative score for each dimension is the total sleep quality score, which ranges from 0 to 21. A total score ≤ 7 indicates good sleep quality, and a total score > 7 indicates poor sleep quality (27).The folded half reliability, Cronbach’s α, and retest reliability of the Chinese version of PSQI are 0.824, 0.845, and 0.994 respectively, which have good reliability and validity (28). In this study, the scale was used for two purposes: first, to describe the prevalence of poor sleep quality (defined as a global PSQI score > 7); and second, to analyze the association between IDH frequency and the PSQI total score as a continuous dependent variable.

### 2.5 Statistical analysis

The data were analyzed using SPSS25.0 statistical software. The test level was set at two-sided of 0.05. Descriptive analysis was performed on the general characteristics of the patients and the scores of each scale. The measurement data that conformed to the normal distribution were described by mean ± standard deviation (*SD*), and the inter-group comparison was performed by two independent sample t-tests. If they did not conform to the normal distribution, the median and interquartile range [*M*(*P*_25_, *P*_75_)] were used for description, and the inter-group comparison was performed by non-parametric tests. The count data were described by frequency (percentage) [n(%)], and the inter-group comparison was performed by chi-square test. Multiple linear regression was used to analyze the relationship between the IDH grouping of patients and anxiety, depression, and sleep quality of MHD patients. Missing values in covariates were handled using multiple imputation with five imputed datasets. Outcome variables were not imputed.

## 3 Results

### 3.1 General characteristics, IDH, anxiety, depression, and sleep quality of MHD patients

#### 3.1.1 General characteristics of MHD patients

This study finally included 467 MHD patients, with an mean age of 54.5 (SD = 13.7) years, 61.5% (n=287) were male, the mean BMI was 22.1 ± 3.6kg/m^2^, and the median dialysis vintage was 64.0 (39.0, 106.0) months. See **Table 1** for details.

**Table 1:** Basic characteristics of maintenance hemodialysis patients in occasional IDH group and frequent IDH group.

Variable	Total(n = 467)	Occasional IDH group (*n* = 344)	Frequent IDH group *n* = 123)	Test statistic (*t/χ^2^/Z*)	P value
	n(%)/Mean ± SD/Median (P_25_, P_75_)	n(%)/Mean ± SD/Median (P_25_, P_75_)	n(%)/Mean ± SD/Median (P_25_, P_75_)		
Sex				7.387	0.007
Male	287 (61.5)	224 (65.1)	63 (51.2)		
Female	180 (38.5)	120 (34.9)	60 (48.8)		
Age (year)	54.5 (13.7)	54 (13.8)	55.7 (13.5)	-1.175	0.241
Height (cm)	165.8 (8.3)	166.8 (8.0)	163 (8.4)	4.429	< 0.001
Dry weight (kg)	60.9 (12.2)	61.7 (12.3)	58.7 (12)	2.311	0.021
BMI (kg/m^2^)	22.1 (3.6)	22.1 (3.5)	22 (3.9)	0.087	0.931
Dialysis age (month)	64.0 (39.0, 106.0)	59.0 (35.0, 93.3)	80.0(49.0, 138.0)	-4.260	< 0.001
Marital status				0.292	0.589
Married	398 (85.2)	295 (85.8)	103 (83.7)		
Others ^a^	69 (14.8)	49 (14.2)	20 (16.3)		
Educational level				2.470	0.116
Junior high school and below	245 (52.5)	173 (50.3)	72 (58.5)		
High school and above	222 (47.5)	171 (49.7)	51 (41.5)		
Working status				5.642	0.018
Retired/Unemployed	325 (69.6)	229 (66.6)	96 (78.0)		
Full-time/Part-time	142 (30.4)	115 (33.4)	27 (22.0)		
Average monthly income*				0.844	0.358
≤3000, China Yuan	226 (53.2)	165 (51.9)	61 (57.0)		
>3000, China Yuan	199 (46.8)	153 (48.1)	46 (43.0)		
Smoking*				0.013	0.910
No	393 (85.4)	290 (85.5)	103(85.1)		
Yes	67 (14.6)	49 (14.5)	18 (14.9)		
Dialysis method				0.744	0.388
HD/HD+HDF	384 (82.2)	286 (83.1)	98 (79.7)		
HD+HDF+HP	83 (17.8)	58(16.9)	25 (20.3)		
Number of medications*				0.037	0.847
≤ 4	280 (60.2)	208 (61.0)	72(60.0)		
≥ 5	181 (39.8)	133 (39.0)	48(40.0)		
Number of comorbidities ^b^*				6.483	0.011
≤ 1	278 (60.0)	216 (63.5)	62 (50.4)		
≥ 2	185 (40.0)	124 (36.5)	61 (49.6)		
Primary disease of ESRD^*^				5.357	0.021
Primary glomerular disease	204 (44.0)	139 (40.8)	65 (52.8)		
Others ^c^	260 (56.0)	202 (59.2)	58 (47.2)		
Hemoglobin (g/L)	111.7 (15.2)	110.6 (15.3)	114.9 (14.4)	-2.732	0.007

BMI, body mass index; HD, hemodialysis; HDF, hemofiltration; HP, hemoperfusion; ESRD, end-stage renal disease.

a included single, widowed, divorced.

b include myocardial infarction, ischemic heart disease, heart failure, arrhythmia, diabetes, cerebrovascular accident, etc.

c included hypertensive nephropathy, diabetic nephropathy, polycystic kidney disease, etc.

*There are missing values due to the following reasons: patients refused to disclose (average monthly income, current smoking status) and the electronic information system was missing (number of medications, total number of comorbidities, primary ESRD disease).

#### 3.1.2 Current status of IDH occurrence

A total of 467 MHD patients with 16,812 hemodialysis sessions were included in this study. The number of IDH occurrences was 1,974 (11.7%) among the total dialysis cases. There were 123 (26.3%) and 344 (73.7%) MHD patients in the frequent IDH group and occasional IDH group, respectively.

#### 3.1.3 Comparison of basic characteristics of MHD patients in different IDH groups

Intergroup comparison of MHD patients with different IDH frequency showed that there were significant statistical differences in gender, height, dry weight, dialysis age, work status, total number of comorbidities, ESRD primary disease, and hemoglobin between the occasional IDH group (n = 344) and the frequent IDH group (n = 123). Patients in the frequent IDH group had lower height, lower dry weight, longer dialysis age, and higher hemoglobin levels. See **Table 1** for details.

#### 3.1.4 Current status of anxiety, depression, and sleep quality in MHD patients

The results indicated an overall anxiety prevalence of 30.4% among MHD patients. The median anxiety score was 2.0 (IQR 0.0, 5.0). Of the participants, 325 (69.6%) were classified as non-anxious, 117 (25.1%) as having mild anxiety, 20 (4.3%) as having moderate anxiety, and 5 (1.1%) as having severe anxiety.

The overall prevalence of depression was 44.3%, with a median depression score of 4.0 (IQR 1.0, 8.0). The depression group comprised 260 individuals (55.7%) without depression, 149 (31.9%) with mild depression, 44 (9.4%) with moderate depression, 10 (2.1%) with moderate-to-severe depression, and 4 (0.9%) with severe depression.

Regarding poor sleep quality, the overall prevalence was 39.9%. The median sleep quality score was 6.0 (IQR 4.0, 10.0). A total of 274 participants (60.1%) were in the good sleep quality group, while 182 (39.9%) were in the poor sleep quality group. The scores of each group are shown in **Table 2**.

**Table 2:** Anxiety, depression and sleep quality in maintenance hemodialysis patients.

Variable	Number of participants *n (%)*	Score *median* (*P*_25_, *P*_75_)	Score mean ± SD
Anxiety conditions ^a^ (*n* = 467)		2.0 (0.0, 5.0)	3.0 ± 3.7
Without anxiety	325 (69.6)	0.0 (0.0, 2.0)	1.0 ± 1.3
Anxiety	142 (30.4)	7.0 (6.0, 8.0)	7.6 ± 3.1
Mild anxiety	117 (25.1)	7.0 (6.0, 7.0)	6.5 ± 1.1
Moderate anxiety	20 (4.3)	10.5 (10.0, 13.0)	11.4 ± 1.6
Severe anxiety	5 (1.1)	19.0 (18.5, 20.5)	19.4 ± 1.1
Depressive condition ^b^ (*n* = 467)		4.0 (1.0, 8.0)	4.8 ± 4.5
Without depression	260 (55.7)	1.0 (0.0, 3.0)	1.5 ± 1.5
Depression	207 (44.3)	8.0 (6.0, 10.0)	8.9 ± 3.5
Mild depression	149 (31.9)	7.0 (6.0, 8.0)	7.1 ± 1.4
Moderate depression	44 (9.4)	12.0 (10.0, 13.0)	11.8 ± 1.4
Moderate to severe depression	10 (2.1)	16.5 (15.0, 18.3)	16.7 ± 1.7
Severe depression	4 (0.9)	22.0 (21.3, 23.5)	22.3 ± 1.3
Sleep quality ^c*^ (*n* = 456)		6.0 (4.0,10.0)	7.0 ± 4.3
Good sleep quality (PSQI ≤ 7)	274 (60.1)	4.0 (3.0, 5.0)	4.0 ± 1.9
Poor sleep quality (PSQI > 7)	182 (39.9)	11.0 (9.0, 14.0)	11.5 ± 3.0

a Measured by Generalized Anxiety Disorder Scale-7.

b Measured by Patient Health Questionnaire-9.

c Measured by Pittsburgh Sleep Quality Index.

*There are missing values.

### 3.2 The relationship between the occurrence of IDH and anxiety, depression and sleep quality in MHD patients

#### 3.2.1 Comparison of anxiety, depression and sleep quality among MHD patients with different basic conditions

Mann-Whitney U test was used to compare anxiety, depression and sleep quality of MHD patients with different basic conditions. The results showed that patients in the frequent IDH group had higher depression scores and more severe depression; patients in the frequent IDH group, retired/unemployed, taking ≥ 5 medications, and having a total of ≥ 2 comorbidities had higher sleep quality scores and worse sleep quality. See **Table 3** for details.

**Table 3:** Comparison of anxiety, depression and sleep quality among different maintenance hemodialysis patients [*M* (*P*_25_, *P*_75_)].

Variable	Anxiety score ^a^ (*n = 467*)	Depression score ^b^ (*n = 467*)	Sleep quality score ^c*^ (*n = 456*)
IDH Groups			
Occasional IDH group	2.0 (0.0, 5.0)	4.0 (1.0, 7.0)	5.0 (4.0, 9.0)
Frequent IDH group	2.0 (0.0, 6.0)	5.0 (1.0, 9.0)	7.0 (4.0, 11.0)
Z	-1.350	-2.473	-3.034
P	0.177	0.013	0.002
Sex			
Male	2.0 (0.0, 6.0)	4.0 (1.0, 8.0)	6.0 (4.0, 10.0)
Female	2.0 (0.0, 5.8)	4.0 (1.0, 8.0)	6.0 (3.0, 10.0)
Z	-0.087	-0.206	-0.995
P	0.930	0.837	0.320
Age			
≤ 60	2.0 (0.0, 6.0)	4.0 (1.0, 8.0)	6.0 (4.0, 10.0)
> 60	1.0 (0.0, 5.0)	4.0 (1.0, 8.0)	7.0 (4.0, 11.0)
Z	-1.328	-0.343	-1.092
P	0.184	0.731	0.275
Height (cm)			
≤ 165	2.0 (0.0, 5.0)	4.0 (1.0, 8.0)	6.0 (4.0, 10.0)
> 165	2.0 (0.0, 6.0)	4.0 (1.0, 8.0)	6.0 (4.0, 10.0)
Z	-0.203	-0.283	-0.540
P	0.839	0.778	0.589
Dry weight (kg)			
≤ 60	2.0 (0.0, 6.0)	4.0 (1.0, 8.0)	6.0 (4.0, 10.0)
> 60	1.0 (0.0, 5.0)	3.5 (1.0, 7.0)	6.0 (4.0, 10.0)
Z	-2.215	-1.415	-0.718
P	0.027	0.157	0.473
BMI (kg/m^2^)			
< 18.5 or > 23.9	1.0 (0.0, 5.0)	4.0 (0.0, 8.0)	6.0 (4.0, 10.0)
18.5 ~ 23.9	2.0 (0.0, 6.0)	4.0 (1.0, 8.0)	6.0 (4.0, 10.0)
Z	-1.444	-1.028	-0.277
P	0.149	0.304	0.782
Dialysis age (month)			
≤ 60	2.0 (0.0, 5.0)	4.0 (1.0, 8.0)	6.0 (4.0, 10.0)
> 60	1.5 (0.0, 5.0)	4.0 (1.0, 8.0)	6.0 (4.0, 10.0)
Z	-0.408	-0.471	-0.598
P	0.683	0.638	0.550
Marital status			
Married	2.0 (0.0, 6.0)	4.0 (1.0, 8.0)	6.0 (4.0, 10.0)
Others^d^	1.0 (0.0, 4.5)	4.0 (0.0, 7.0)	5.0 (4.0, 9.0)
Z	-1.187	-0.812	-0.299
P	0.235	0.417	0.765
Educational level			
Junior high school and below	2.0 (0.0, 5.0)	4.0 (1.0, 8.0)	7.0 (4.0, 10.0)
High school and above	1.5 (0.0, 5.0)	4.0 (0.0, 7.0)	5.0 (4.0, 9.0)
Z	-0.291	-0.527	-1.767
P	0.771	0.598	0.077
Working status			
Retired/Unemployed	2.0 (0.0, 6.0)	4.0 (1.0, 8.0)	7.0 (4.0, 10.0)
Full-time/Part-time	2.0 (0.0, 5.0)	4.0 (1.0, 7.0)	5.0 (4.0, 9.0)
Z	-0.343	-0.451	-2.145
P	0.732	0.652	0.032
Average monthly income			
≤ 3000, China Yuan	2.0 (0.0, 6.0)	4.0 (1.0, 8.0)	6.0 (4.0, 10.0)
> 3000, China Yuan	1.5 (0.0, 5.0)	3.0 (1.0, 7.0)	6.0 (4.0, 10.0)
Z	-1.751	-1.329	-1.117
P	0.080	0.184	0.264
Smoking			
No	2.0 (0.0, 5.0)	4.0 (1.0, 8.0)	6.0 (4.0, 10.0)
Yes	2.0 (0.0, 6.0)	5.0 (1.0, 8.0)	7.5 (4.0, 11.0)
Z	-0.013	-0.940	-1.211
P	0.990	0.347	0.226
Dialysis method			
HD/HD+HDF	2.0 (0.0, 5.0)	4.0 (1.0, 8.0)	6.0 (4.0, 10.0)
HD+HDF+HP	2.0 (0.0, 6.0)	4.0 (1.0, 8.0)	7.0 (4.0, 11.0)
Z	-0.730	-1.157	-1.751
P	0.465	0.247	0.080
Number of medications			
≤ 4	2.0 (0.0, 5.0)	4.0 (1.0, 8.0)	5.0 (3.0, 9.0)
≥ 5	1.0 (0.0, 6.0)	4.0 (1.0, 7.0)	7.0 (4.0, 11.0)
Z	-0.100	-0.646	-3.515
P	0.921	0.518	< 0.001
Number of comorbidities ^e^			
≤ 1	2.0 (0.0, 5.0)	4.0 (1.0, 8.0)	5.0 (3.0, 9.0)
≥ 2	2.0 (0.0, 6.0)	4.0 (1.0, 7.0)	7.0 (4.0, 11.0)
Z	-0.522	-0.155	-3.089
P	0.602	0.877	0.002
Primary disease of ESRD			
Primary glomerular disease	1.5 (0.0, 5.0)	4.0 (1.0, 7.0)	7.0 (4.0, 11.0)
Others ^f^	2.0 (0.0, 5.8)	4.0 (1.0, 8.0)	6.0 (4.0, 9.0)
Z	-0.387	-0.997	-1.343
P	0.699	0.319	0.179
Hemoglobin ^g^ (g/L)			
normal	2.0 (0.0, 6.0)	4.0 (1.0, 8.0)	6.0 (3.0, 10.0)
abnormal	2.0 (0.0, 5.0)	4.0 (1.0, 7.0)	6.0 (4.0, 9.0)
Z	-1.264	-1.276	-0.147
P	0.206	0.202	0.883

BMI, body mass index; HD, hemodialysis; HDF, hemofiltration; HP, hemoperfusion; ESRD, end-stage renal disease.

a Measured by Generalized Anxiety Disorder Scale-7.

b Measured by Patient Health Questionnaire-9.

c Measured by Pittsburgh Sleep Quality Index.

d included single, widowed, divorced.

e include myocardial infarction, ischemic heart disease, heart failure, arrhythmia, diabetes, cerebrovascular accident, etc.

f included hypertensive nephropathy, diabetic nephropathy, polycystic kidney disease, etc.

g Normal hemoglobin value: 120-160g/L for men and 110-150g/L for women, otherwise it is abnormal.

*There are missing values.

#### 3.2.2 Relationship between IDH groups and anxiety, depression and sleep quality in MHD patients

This study used anxiety, depression, and sleep quality scores as dependent variables and IDH grouping as independent variables and used multivariate linear regression to establish a model. In order to verify the reliability of the model results, this study included stratification into the patient’s basic information as a covariate to explore the relationship between IDH grouping and anxiety, depression, and sleep quality in MHD patients. First, model 1 was established without correcting any confounding factors. Then, based on model 1, the related factors that were different between the groups and the potential related factors mentioned in previous literature (29, 30) were included in model 2 and model 3 for multivariate linear regression analysis. Model 2 corrected the confounding factors of gender, age, height, dry weight, education level, work status, average monthly income, and current smoking. Model 3 corrected the confounding factors of gender, age, height, dry weight, education level, work status, average monthly income, current smoking, dialysis age, dialysis method, number of medications, total number of comorbidities, ESRD primary disease, and hemoglobin.

The results showed that IDH grouping was significantly associated with anxiety, depression, and sleep quality scores (*P* < 0.05). Compared with the occasional IDH group, the anxiety score of the frequent IDH group was significantly higher by 1.279 points (β = 1.279, 95%CI = 0.489 ~ 2.068, *P* = 0.002), the depression score was significantly higher by 1.853 points (β = 1.853, 95%CI = 0.895 ~ 2.812, *P* < 0.001), and the sleep quality score was significantly higher by 1.215 points (β = 1.215, 95%CI = 0.294 ~ 2.136, *P* = 0.010). The results of this study showed that the frequent IDH group had higher anxiety, depression, and sleep quality scores, and more severe anxiety, depression, and poor sleep quality than the occasional IDH group. See **Table 4** for details.

**Table 4:** Linear regression model of the relationship between IDH groups and anxiety, depression, and sleep quality.

Variable	Model 1			Model 2			Model 3		
	*R^2^*	*β (95%CI)*	*P*	*R^2^*	*β* (95%CI)	*P*	*R^2^*	*β* (95%CI)	*P*
Anxiety (*n* = 467)	0.023	1.252 (0.506 ~ 1.999)	0.001	0.041	1.267 (0.500 ~ 2.033)	0.001	0.059	1.279 (0.489 ~ 2.068)	0.002
Depression (*n* = 467)	0.030	1.767 (0.859 ~2.675)	< 0.001	0.048	1.729 (0.795 ~ 2.644)	< 0.001	0.034	1.853 (0.895 ~ 2.812)	< 0.001
Sleep quality (*n* = 456)	0.026	1.594 (0.699 ~ 2.490)	< 0.001	0.056	1.466 (0.550 ~ 2.382)	0.002	0.140	1.215 (0.294 ~ 2.136)	0.010

Model 1 did not adjust for any factors.

Model 2 adjusted for gender, age, height, dry weight, education level, work status, average monthly income, and current smoking;.

Model 3 adjusted for gender, age, height, dry weight, education level, work status, average monthly income, current smoking, dialysis age, dialysis method, dialysis frequency, number of medications, total number of comorbidities, primary ESRD disease, and hemoglobin.

## 4 Discussion

IDH represents a frequent complication among patients on MHD, significantly compromising dialysis adequacy and overall clinical outcomes. It is associated with serious adverse events, including increased cardiovascular morbidity and mortality (31, 32), as well as reduced quality of life (QOL) (33). In recent years, psychological factors have gained recognition as important moderators of clinical outcomes and QOL in chronic disease populations. However, the relationship between IDH and psychological comorbidities—such as anxiety, depression, and sleep disturbances—remains insufficiently examined. This study aimed to investigate the association between IDH and these psychological and sleep-related indicators, with the goal of informing more holistic interventional approaches for affected patients.

Our analysis included 16,812 dialysis sessions from 467 MHD patients and revealed an IDH incidence of 11.7% per session, with 26.3% of patients classified into the frequent IDH group. These figures align with a previous cross-sectional study of 545 patients and 3,261 sessions, which reported an IDH incidence of 14.4% (34). Significant differences were observed between the frequent and occasional IDH groups regarding sex, height, dry weight, dialysis age, working status, number of comorbidities, primary disease of ESRD, and hemoglobin level. Specifically, females, patients with lower height or dry weight, longer dialysis vintage, retired or unemployed status, presence of ≥ 2 comorbidities, and those with glomerular disease as the primary etiology were more likely to experience frequent IDH—consistent with earlier findings (33). Notably, the higher IDH frequency in female patients observed in this study, combined with previous literature clearly documenting a significantly higher diagnostic rate of anxiety, depression, and stress-related disorders in females than in males (35, 36), raises the possibility that sex-related differences in psychological characteristics could contribute to the observed disparity, though direct evidence is lacking in this study. However, due to the limited sample size, robust statistical inference regarding how sex moderates the relationship between IDH and mood disorders could not be made in this study, and thus the above hypothesis awaits validation in future research. Future studies employing larger cohort samples and incorporating systematic psychological and sleep assessments are warranted to determine whether the associations between IDH and these psychological/behavioral factors differ by sex.

In this study, 30.4% and 44.3% of the total MHD sample exhibited anxiety and depression symptoms, respectively, ranging from mild to severe. This prevalence is consistent with prior reports highlighting a substantial psychological distress burden among MHD patients (37, 38). Comparative analysis further indicated that patients with frequent IDH showed more pronounced depressive symptoms and a trend toward higher anxiety. These results emphasize the need for clinicians to strengthen psychological monitoring and supportive care in this subgroup to mitigate mood deterioration. Regarding sleep quality, the prevalence of sleep disturbances in our study was 39.9%, notably lower than the 86.6% reported by Masoumi et al. (39). This discrepancy may stem from methodological differences, including the use of a 5−point sleep disturbance scale, inclusion of patients with longer dialysis vintage, and enrollment of both hemodialysis and peritoneal dialysis populations in the latter study. Furthermore, intergroup comparisons revealed significantly poorer sleep quality among frequent IDH patients, those who were retired or unemployed, those taking ≥ 5 medications, and those with ≥ 2 comorbidities. Thus, early targeted sleep assessment and structured health management interventions are clinically warranted in these high−risk groups.

Multivariable linear regression showed that frequent IDH was independently associated with more severe anxiety and depression symptoms, as well as poorer sleep quality. To assess the clinical relevance of these associations, we referred to the concept of the Minimum Clinically Important Difference (MCID). For anxiety and depression, a longitudinal primary care study estimated MCIDs of approximately 1.5 points on the GAD−7 and 1.7 points on the PHQ−9 as corresponding to a noticeable improvement in well−being (40). In our study, 30.4% and 44.3% of participants exhibited clinically significant anxiety (GAD−7 ≥ 5) and depression (PHQ−9 ≥ 5). Against this background, the between−group differences observed—1.279 points on the GAD−7 and 1.853 points on the PHQ−9—closely approximate these MCID thresholds, suggesting that the differences may be not only statistically significant but also clinically perceptible. Regarding sleep quality, a prior study (41) derived MCID estimates for the Pittsburgh Sleep Quality Index (PSQI) ranging from approximately 1.3 to 3.5 points, with the lower bound (1.3–1.5 points) reflecting a meaningful patient−reported improvement. In our analysis, the frequent IDH group had PSQI scores 1.215 points higher than the occasional IDH group, approaching the lower end of the reported MCID range and further supporting potential clinical relevance. Collectively, these findings suggest that the worsening of anxiety, depression, and sleep quality associated with frequent IDH likely reflects changes meaningful to patients and worthy of clinical attention.

Given that patients with frequent IDH exhibited more severe depressive symptoms (with a PHQ-9 difference approaching the MCID) and poorer sleep quality (with a PSQI difference approaching the lower bound of the MCID), this psycho-physiological association warrants further exploration. It should be emphasized that, owing to the cross-sectional design of this study, no causal inferences can be drawn. A bidirectional interaction is plausible: on one hand, IDH may adversely affect patients’ mental status and sleep through hemodynamic abnormalities (e.g., myocardial strain, cerebral hypoperfusion, intestinal ischemia), loss of residual kidney function, and high symptom burden (42); on the other hand, emotional and sleep disturbances may influence blood pressure regulation via neuroendocrine pathways, thereby increasing the risk of hypotension (43). From a mechanistic perspective, recurrent hemodynamic instability during dialysis may activate stress-response systems, including the hypothalamic–pituitary–adrenal axis and autonomic nervous system (44). Alterations in cortisol secretion and autonomic regulation have been linked to both blood pressure instability and psychological symptoms (45). These pathways may provide a plausible biological framework for understanding the association observed in this study; however, they were not directly measured and should be interpreted as hypotheses for future research.

Based on these findings, we recommend routine psychological and sleep assessments using the GAD-7, PHQ-9, and PSQI in MHD patients, particularly those with frequent IDH, retired/unemployed status, use of five or more medications, or the presence of two or more comorbidities. For patients screening positive, non-pharmacological interventions combined with dialysis regimen optimization may be considered.

### 4.1 Limitation

This study has several limitations. First, IDH was defined solely by the nadir systolic blood pressure during dialysis (<100 mmHg), without incorporating documentation of clinical symptoms or interventions. This simplified definition may lead to an underestimation of clinically significant IDH and could attenuate the observed associations with patient-reported outcomes. Future research should adopt a more comprehensive IDH criterion and examine how different definitions influence emotional well-being and sleep quality. Second, our study did not analyze the associations between preexisting psychiatric diagnoses and IDH frequency, anxiety, depression, or sleep quality, which may constitute a potential residual confounding factor. Future prospective studies should include systematic baseline screening for psychiatric history to better differentiate between preexisting mental disorders and symptoms that emerge during MHD treatment. Third, medication type was not included as an independent variable in our analysis. Consequently, the impact of drugs with different pharmacological mechanisms on mood and sleep may not be fully captured by the single metric of “number of medications”. Future research should systematically record detailed medication regimens to clarify the independent effects and potential interactions of specific drug classes. Finally, this work was primarily observational and did not investigate the underlying pathophysiological mechanisms, such as those involving inflammatory markers or autonomic nervous system activity. Prospective studies incorporating biomarker assessments are needed to elucidate the causal pathways linking IDH with psychological and sleep disturbances.

## 5 Conclusion

Patients with MHD exhibit a high incidence of IDH, along with varying degrees of anxiety, depression, and sleep disturbances. Notably, a significant correlation has been observed between IDH occurrence and the severity of anxiety, depression, and poor sleep quality. However, given the lack of causal evidence, future research should employ longitudinal study designs to further validate these associations and investigate potential underlying mechanisms, thereby providing more robust evidence to guide clinical practice.

## Data Availability

The raw data supporting the conclusions of this article will be made available by the authors, without undue reservation.
